# Mixed Infections of Four Viruses, the Incidence and Phylogenetic Relationships of Sweet Potato Chlorotic Fleck Virus (Betaflexiviridae) Isolates in Wild Species and Sweetpotatoes in Uganda and Evidence of Distinct Isolates in East Africa

**DOI:** 10.1371/journal.pone.0167769

**Published:** 2016-12-22

**Authors:** Arthur K. Tugume, Settumba B. Mukasa, Jari P. T. Valkonen

**Affiliations:** 1 Department of Agricultural Sciences, Faculty of Agriculture and Forestry, University of Helsinki, Helsinki, Finland; 2 Department of Plant Sciences, Microbiology and Biotechnology, School of Biosciences, College of Natural Sciences, Makerere University, Kampala, Uganda; 3 Department of Agricultural Production, School of Agricultural Sciences, College of Agricultural and Environmental Sciences, Makerere University, Kampala, Uganda; Oklahoma State University, UNITED STATES

## Abstract

Viruses infecting wild flora may have a significant negative impact on nearby crops, and *vice-versa*. Only limited information is available on wild species able to host economically important viruses that infect sweetpotatoes (*Ipomoea batatas*). In this study, *Sweet potato chlorotic fleck virus* (SPCFV; *Carlavirus*, Betaflexiviridae) and *Sweet potato chlorotic stunt virus* (SPCSV; *Crinivirus*, Closteroviridae) were surveyed in wild plants of family Convolvulaceae (genera *Astripomoea*, *Ipomoea*, *Hewittia* and *Lepistemon*) in Uganda. Plants belonging to 26 wild species, including annuals, biannuals and perennials from four agro-ecological zones, were observed for virus-like symptoms in 2004 and 2007 and sampled for virus testing. SPCFV was detected in 84 (2.9%) of 2864 plants tested from 17 species. SPCSV was detected in 66 (5.4%) of the 1224 plants from 12 species sampled in 2007. Some SPCSV-infected plants were also infected with *Sweet potato feathery mottle virus* (SPFMV; *Potyvirus*, Potyviridae; 1.3%), *Sweet potato mild mottle virus* (SPMMV; *Ipomovirus*, Potyviridae; 0.5%) or both (0.4%), but none of these three viruses were detected in SPCFV-infected plants. Co-infection of SPFMV with SPMMV was detected in 1.2% of plants sampled. Virus-like symptoms were observed in 367 wild plants (12.8%), of which 42 plants (11.4%) were negative for the viruses tested. Almost all (92.4%) the 419 sweetpotato plants sampled from fields close to the tested wild plants displayed virus-like symptoms, and 87.1% were infected with one or more of the four viruses. Phylogenetic and evolutionary analyses of the 3′-proximal genomic region of SPCFV, including the silencing suppressor (NaBP)- and coat protein (CP)-coding regions implicated strong purifying selection on the *CP* and *NaBP*, and that the SPCFV strains from East Africa are distinguishable from those from other continents. However, the strains from wild species and sweetpotato were indistinguishable, suggesting reciprocal movement of SPCFV between wild and cultivated Convolvulaceae plants in the field.

## Introduction

There is evidence that wild flora acts as a reservoir of viruses causing significant losses in nearby crops and vice versa [1–7]. However, information about viruses in wild species is still quite limited. This may in part be due to the fact that viral infections in wild plants are often symptomless, even when the same infection may have obvious symptoms in cultivated plants [8–10]. Whether the same virus strains can infect wild and cultivated plants, but are better adapted to wild plants and hence cause no symptoms, is an issue requiring further study [11, 12]. The geospatial distribution and genetic variability of viruses in wild species is also poorly understood [13, 14]. Although some metagenomic surveys have explored virus diversity in wild plant communities [14–19], only a few studies have described the genetic variability of individual virus species in wild plants in relation to isolates found in cultivated plants [20–27]. Moreover, few studies have compared isolates of plant viruses from wild and cultivated hosts across broad geographical areas [22–24, 28–30]. Thus, studies comparing virus populations in weeds or wild species and crop species that share an agro-ecological interface are needed to gain insights into the evolutionary and ecological dynamics of plant virus populations, which in turn are needed to facilitate plant virus disease management [8, 31, 32].

The incidence and impact of plant viruses at the agro-ecological interface are often exacerbated in evergreen tropical environments, where susceptible cultivated and wild plants are continuously available, providing the necessary environment for viral replication and vectors for viral transmission [30, 33, 34]. Plant virus diseases not only have an economic impact but also may cause starvation, especially when the cultivated host plant constitutes a ‘food security’ crop [35–38]. An example is the sweetpotato, *Ipomoea batatas* (L.) Lam., the world’s third-most-important root crop and a critical food security crop in sub-Saharan Africa [38–40]. Globally, over 30 viruses are known to infect sweetpotatoes [41–43].

Sweetpotatoes are grown as a perennial crop in local cropping systems in Uganda and elsewhere in East Africa. Sources of healthy planting materials are limited [44, 45]. Perreniality and lack of healthy sweetpotato planting materials coupled with the abundance of insect vectors transmitting the viruses promotes yield losses due to virus diseases [46]. The most severe yield losses occur in sweetpotato plants co-infected with the whitefly-transmitted *Sweet potato chlorotic stunt virus* (SPCSV; genus *Crinivirus*, family Closteroviridae) and the aphid-transmitted *Sweet potato feathery mottle virus* (SPFMV; genus *Potyvirus*, family Potyviridae). Co-infection with these viruses results in so-called Sweet potato virus disease (SPVD), characterized by leaf malformation, stunted plants and nearly complete loss of yields [47–50]. Similar but milder symptoms develop in sweetpotato plants co-infected with SPCSV and *Sweet potato chlorotic fleck virus* (SPCFV; genus *Carlavirus*, family Betaflexiviridae), *Sweet potato mild mottle virus* (SPMMV; genus *Ipomovirus*; family Potyviridae) [49, 51] or sweepoviruses (genus *Begomovirus*, family Geminiviridae) [52]. The frequent co-infection of sweetpotatoes with SPCFV and SPFMV suggests that these viruses may be transmitted by a common vector [53, 54], but the vector of SPCFV remains to be identified [55]. Whiteflies transmit sweepoviruses and were also initially reported as vectors of SPMMV [56], but these results could not be confirmed in later studies [55].

Previous studies in Uganda have shown that SPFMV, SPMMV and SPCSV from wild plants of the family Convolvulaceae are phylogenetically similar to those found in cultivated sweetpotatoes [22–24]. SPFMV and SPMMV, respectively, were detected in 24 and 21 wild plant species and in 23 and 20 districts, respectively, surveyed in the country [23, 57]. Furthermore, 12 wild Convolvulaceae species were found to be infected with SPCSV [24], but the geographical distribution of SPCSV in wild vegetation in Uganda and the wild host species and co-infection of SPCSV with other viruses in wild plants were not reported. Similarly, information regarding SPCFV infection in wild plants of Convolulacea is lacking, even though SPCFV occurs sweetpotatoes in Uganda [58] and other East African countries such as Kenya [53, 59], Tanzania [60] and Rwanda [61], as well as western Africa [62], Asia, Australia, East Timor and Latin America [54, 63–66].

The aim of this study was to determine the incidence of SPCFV and SPCSV and their rates of co-infection with SPFMV and SPMMV, in wild species interfacing with cultivated sweetpotatoes in the major agro-ecological zones of Uganda, and to study the genetic variability of SPCFV.

## Results

### Virus-like symptoms in wild plants

**Fig 1:**
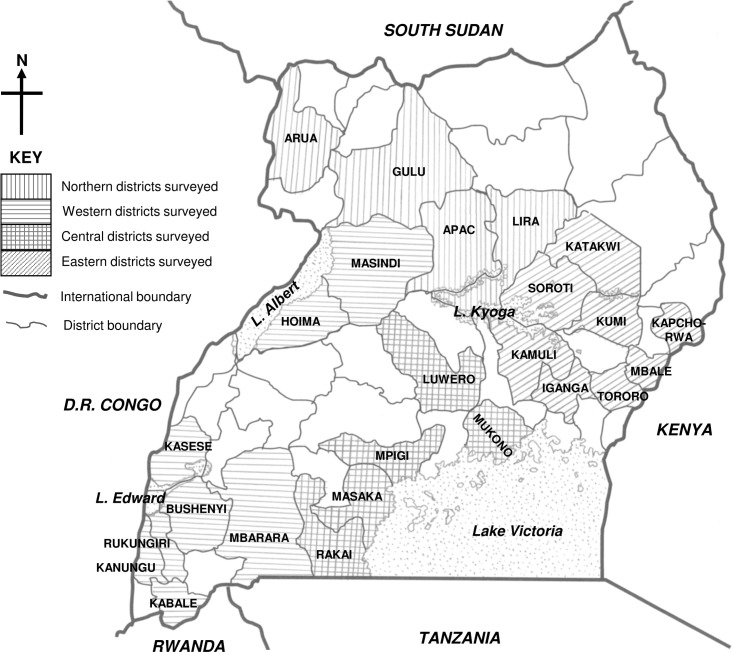
Map of Uganda showing the districts surveyed for wild Convolvulaceae species and viruses in Uganda.

**Fig 2:**
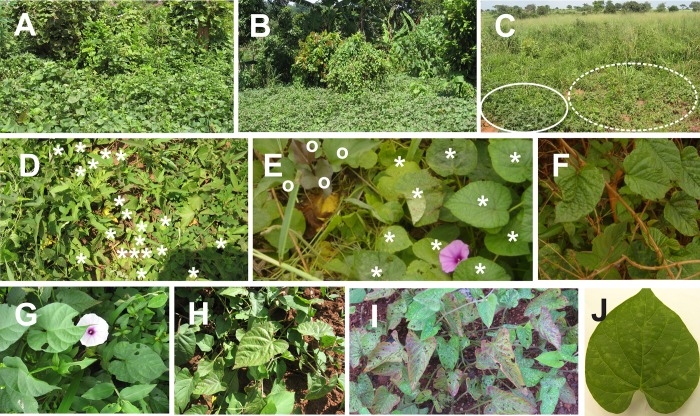
Examples of wild species of Convolvulaceae in their natural habitats in Uganda, and some virus-like symptoms. (**A**) *Ipomoea wightii* and (**B**) *I*. *acuminata* (in the background) trailing into sweet potato field (foreground) in the Mbarara and Mukono districts, respectively. Wild vegetation in these districts is dominated by tall shrubs. (**C**) *I*. *sinensis* (dotted circle) in close proximity to sweetpotato field (edge inside solid circle) in the Soroti district, which is dominated by short grassland vegetation (background). (**D**) *I*. *sinensis* (white asterisks) growing as weeds in a sweetpotato field in the Katakwi district. (**E**) Sweetpotato plant (white **o**) mixed with plants of *I*. *wightii* (white asterisks) in the Mukono district. (**F**-**J**) Examples of virus-like symptoms. (**F**) Leaf chlorosis in *H*. *sublobata*. (**G**) Chlorotic spots on a leaf of *I*. *tenuirostris*. (**H, I**) Mild (**H**) and severe (**I**) purpling in old leaves of *I*. *sinensis*. (**J**) Mild chlorotic spots on a leaf of *I*. *acuminata*. Plants in **F**, **G** and **J** tested positive for SPCFV; plants in **H** and **I** tested positive for SPCSV.

A total of 2864 wild plants of the family Convolvulaceae (genera *Astripomoea*, *Hewittia*, *Ipomoea* and *Lepistemon*) were sampled from their natural habitats in four agro-ecological zones in Uganda where sweetpotato crops are grown (Figs 1 and 2, S1 Table). The natural habitats of the wild plants surveyed were in close proximity (within 500 m) to cultivated sweetpotato fields. The wild plants were observed to trail into the sweetpotato fields, especially in the western (Fig 2A), central (Fig 2B) and eastern (Fig 2C) zones. Some wild plants grew as weeds in sweetpotato fields in the eastern zone (Fig 2D). Volunteer sweetpotato plants were found growing among wild plants in the central zone (Fig 2E).

Virus-like symptoms were observed in a total of 367 wild plants (12.8%) collected over the two sampling years (2004 and 2007); of these, 42 plants (11.4%) tested negative for all four viruses. In contrast, 132 (5.3%) of 2497 symptomless wild plants tested positive for at least one of the four viruses. The symptomless but virus-positive wild plants constituted 15.8% of all 836 wild plants that tested positive for at least one virus. In sweetpotatoes, 5 (1.3%) of the 387 plants with symptoms tested negative for all four viruses. On the other hand, 10 (31.3%) of 32 symptomless plants tested positive for at least one virus.

Leaf chlorosis was observed in *H*. *sublobata* (Fig 2F) and chlorotic spots were displayed in *I*. *tenuirostris* (Fig 2G) and *I*. *acuminata* (Fig 2J) infected with SPCFV. Mild to severe purpling of older leaves was observed in plants of *I*. *sinensis* infected with SPCSV (Fig 2H and 2I).

### Incidence of SPCFV in wild plants

Plants showing a consistent and unambiguous positive reaction in three independent NCM-ELISA experiments were deemed SPCFV-infected. SPCFV was detected in 84 (2.9%) of 2864 wild plants tested, including *H*. *sublobata*, *L*. *owariensis* and 15 of the 26 *Ipomoea* species tested (Table 1, S1 Table). All of these 17 wild species of family Convolvulaceae represent previously unknown natural hosts for SPCFV. In eleven species (*I*. *acuminata*, *I*. *cairica*, *I*. *eriocarpa*, *I*. *involucrata*, *I*. *obscura*, *I*. *sinensis*, *I*. *tenuirostris*, *I*. *wightii*, *Astripomoea hyocyamoides*, *H*. *sublobata* and *L*. *owariensis*) from which over 40 plants were tested, the overall incidence of SPCFV ranged from 1.8% in *I*. *tenuirostris* and *I*. *wightii* to 5.2% in *L*. *owariensis* (Table 1). No tested plants of *I*. *cordofana*, *I*. *eriocarpa*, *I*. *fistulosa*, *I*. *grantii*, *I*. *involucrata*, *I*. *polymorpha*, *I*. *spathulata*, *I*. *velutipes*, *A*. *grantii* or *A*. *hyocyamoides* were positive for SPCFV (Table 1). The lowest incidence of SPCFV in wild plants (0.7%) was recorded in the Masindi district in western Uganda, and the highest incidence (9.0%) was found in the Katakwi district in eastern Uganda (Table 1).

**Table 1:** Incidence of *Sweet potato chlorotic fleck virus* in wild plants and cultivated sweetpotato plants from different agro-ecological zones of Uganda.

			Central zone^d^					Northern zone^d^				Eastern zone^d^								Western zone^d^							
Plant species	Life cycle^a^	Total no. of plants^c^	LUW	MKN	MSK	RKI	MPG	LIR	APC	GUL	ARU	KTK	SOR	KUM	MBL	KAP	TOR	KML	IGG	RUK	KNG	KBL	BUS	MBR	KAS	MAS	HOM
*Ipomoea acuminata*	P	157(4.5)	0(16)	0(11)	0(9)	-	0(36)	-	-	-	-	-	-	-	6(35)	-	-	-	-	0(9)	0(22)	-	1(8)	0(6)	-	-	0(5)
*I*. *aquatica*	P	22(9.1)	-	-	-	-	-	-	-	-	-	2(20)	0(2)	-	-	-	-	-	-	-	-	-	-	-	-	-	-
*I*. *blepharophylla*	P	14(7.1)	-	-	-	-	-	-	-	-	-	1(14)	-	-	-	-	-	-	-	-	-	-	-	-	-	-	-
*I*. *cairica*	P	220(4.5)	0(2)	0(6)	1(19)	0(30)	1(19)	-	-	-	-	-	-	-	0(3)	-	0(5)	3(26)	-	1(13)	-	-	1(25)	3(57)	-	0(14)	0(1)
*I*. *cordofana*	A	10(0)	-	-	-	-	-	-	0(10)	-	-	-	-	-	-	-	-	-	-	-	-	-	-	-	-	-	-
*I*. *crepidiformis*	P	30(3.3)	-	-	-	-	-	-	-	-	-	-	0(20)	1(10)	-	-	-	-	-	-	-	-	-	-	-	-	-
*I*. *eriocarpa*	A	134(0)	-	-	-	-	-	0(38)	0(9)	-	-	-	-	-	-	-	-	-	-	-	-	-	-	0(7)	0(47)	0(33)	-
*I*. *fistulosa*	P	25(0)	-	0(25)	-	-	-	-	-	-	-	-	-	-	-	-	-	-	-	-	-	-	-	-	-	-	-
*I*. *grantii*	P	9(0)	-	0(9)	-	-	-	-	-	-	-	-	-	-	-	-	-	-	-	-	-	-	-	-	-	-	-
*I*. *hederifolia*	A	47(2.1)	-	0(11)	0(5)	-	-	-	-	-	-	-	1(4)	-	-	-	-	0(8)	-	0(9)	-	-	-	-	-	-	0(10)
*I*. *hildebrandtii*	P	17(5.9)	-	-	-	0(2)	-	-	-	-	-	-	-	-	0(3)	-	-	0(7)	1(2)	0(1)	0(1)	-	-	0(1)	-	-	-
*I*. *involucrata*	P	78(0)	-	-	-	-	-	-	-	-	-	-	-	0(12)	-	-	-	-	-	-	0(10)	0(50)	0(6)	-	-	-	-
*I*. *obscura*	P	154(4.5)	-	0(6)	0(19)	0(8)	0(6)	-	0(8)	-	-	-	1(18)	1(10)	-	2(32)	-	-	-	0(3)	-	0(1)	0(7)	0(14)	1(10)	0(1)	1(5)
*I*. *polymorpha*	BA	10(0)	-	-	-	-	-	-	-	-	-	-	-	-	-	0(10)	-	-	-	-	-	-	-	-	-	-	-
*I*. *purpurea*	A	34(5.9)	-	-	0(6)	0(2)	-	-	-	-	-	-	-	-	-	-	-	-	-	-	-	-	-	2(16)	-	-	-
*I*. *repens*	P	27(11.1)	-	-	-	-	-	-	-	-	-	-	2(4)	1(14)	0(9)	-	-	-	-	-	-	-	-	-	-	-	-
*I*. *rubens*	P	37(2.7)	-	-	0(2)	0(8)	-	-	-	-	-	1(13)	0(8)	-	-	-	0(1)	-	0(5)	-	-	-	-	-	-	-	-
*I*. *sinensis*	A	374(3.5)	-	-	-	-	-	-	-	0(54)	0(36)	8(64)	2(42)	2(31)	0(16)	0(7)	0(24)	0(23)	1(28)	-	0(7)	-	-	-	-	0(40)	0(2)
*I*. *spathulata*	P	42(0)	-	-	-	-	-	-	-	-	-	-	-	-	-	0(42)	-	-	-	-	-	-	-	-	-	-	-
*I*. *stenobasis*	P	23(8.7)	-	-	-	-	-	-	-	-	0(7)	-	1(7)	-	0(3)	-	-	0(6)	-	-	-	-	-	-	-	-	-
*I*. *tenuirostris*	P	395(1.8)	0(17)	0(28)	-	0(3)	0(13)	0(2)	0(31)	-	-	-	1(7)	-	1(44)	0(60)	0(1)	0(6)	-	1(65)	0(25)	0(18)	3(58)	0(8)	0(7)	1(2)	-
*I*. *velutipes*	A	10(0)	-	-	-	-	-	-	-	-	-	-	-	-	0(2)	-	-	-	-	-	-	-	-	0(8)	-	-	-
*I*. *wightii*	P	113(1.8)	0(9)	0(11)	0(12)	0(26)	0(1)	-	-	-	-	-	-	-	0(2)	-	1(4)	-	-	0(5)	1(18)	0(2)	0(6)	0(15)	-	-	0(2)
*A*. *grantii*	P	42(0)	-	-	0(7)	0(15)	-	-	-	-	-	-	-	-	-	-	-	-	-	-	0(20)	-	-	-	-	-	-
*A*. *hyoscyamoides*	P	64(0)	0(12)	-	-	-	-	-	-	-	-	-	0(5)	-	0(38)	-	-	0(5)	-	0(4)	-	-	-	-	-	-	-
*H*. *sublobata*	P	687(2.8)	0(28)	0(18)	0(45)	0(43)	0(44)	0(22)	0(10)	0(2)	-	-	0(19)	-	1(28)	3(12)	5(86)	4(50)	3(41)	0(22)	1(51)	-	1(40)	1(29)	0(8)	0(48)	0(41)
*L*. *owariensis*	P	97(5.2)	-	0(22)	-	-	0(3)	-	-	-	-	-	0(2)	-	1(16)	-	2(23)	2(25)	-	-	0(2)	-	-	-	-	0(1)	0(3)
Total no. wild plants^b^		2864(2.9)	0(84)	0(147)	1(124)	0(137)	1(122)	0(62)	0(68)	0(56)	0(43)	12(113)	8(138)	5(77)	9(199)	5(163)	8(144)	9(156)	5(76)	2(131)	2(156)	0(71)	6(156)	8(161)	1(72)	1(139)	1(69)
SPCFV incidence (%)			0	0	0.8	0	0.8	0	0	0	0	9.0	5.8	6.5	4.5	3.1	5.6	5.8	6.6	1.5	1.3	0	3.8	4.9	1.4	0.7	1.4
Sweetpotato	P	419(4.1)	-	0(19)	3(40)	1(28)	2(23)	-	-	0(14)	2(21)	0(14)	0(17)	-	1(33)	0(23)	0(16)	1(32)	-	1(26)	2(36)	-	1(21)	0(15)	-	0(16)	3(25)

^a^Lifecycle of species: A, annual; BA, biannual; P, perennial.

^b^Number of SPCFV-positive plants followed (in parentheses) by total number of plants tested per district. The numbers of plants tested in each year (2004 and 2007) are shown in S1 Table.

^c^Total number of plants tested per species followed (in parentheses) by percentage of plants of each species testing positive for SPCFV.

^d^Number of SPCFV-positive plants followed (in parentheses) by number of plants tested per district. ‘─’ indicates that the plant species was not observed in that district. Central region districts (Lake Victoria basin): LUW = Luwero, MKN = Mukono, MSK = Masaka, RKI = Rakai, MPG = Mpigi. Northern region districts: LIR = Lira, APC = Apac, GUL = Gulu, ARU = Arua. Eastern region districts: KTK = Katakwi, SOR = Soroti, KUM = Kumi, MBL = Mbale, KAP = Kapchorwa, TOR = Tororo, KML = Kamuli, IGG = Iganga. Western region districts: RUK = Rukungiri, KNG = Kanungu, KBL = Kabale, BUS = Bushenyi, MBR = Mbarara, KAS = Kasese, MAS = Masindi, HOM = Hoima.

SPCFV was also detected in 17 (4.1%) of 419 cultivated sweetpotato plants sampled from the four agro-ecological zones. SPFMV, SPCSV and SPMMV were detected in 177 (44.6%), 112 (29.5%) and 59 (14.0%), respectively, of the tested sweetpotato plants.

To allow later sequence characterization of its genome, SPCFV isolates from wild plants and sweetpotato plants were mechanically inoculated onto sweetpotato cv. Tanzania and maintained in a greenhouse. Five SPCFV isolates were collected from wild plants, including two from *I*. *acuminata* (Mbale, eastern zone) and one each from *I*. *acuminata* (Bushenyi, western zone), *I*. *cairica* (Mbigi, central zone) and *I*. *tenuirostris* (Masindi, western zone). Four SPCFV isolates were collected from sweetpotato plants, including two from the western zone and one each from the central and northern zones.

### Detection and incidence of SPCSV in wild plants

Positive reaction with SPCSV antibodies was observed in 66 (5.4%) of 1224 wild plants tested in triplicate by triple antibody sadwich-ezyme linked immunosorbent assay (TAS-ELISA) (Table 2). Only 27% of seropositive plants showed virus-like symptoms. SPCSV-infected plants belonged to 10 *Ipomoea* species plus *H*. *sublobata* and *L*. *owariensis* (Table 2). The SPCSV incidence in species from which at least 51 plants were tested was 9.3% in *I*. *obscura*, 8.8% in *I*. *cairica*, 7.8% in *I*. *acuminata*, 5.6% in *I*. *sinensis*, 5.5% in *I*. *tenuirostris* and 2.6% in *H*. *sublobata* (Table 2). Scions from 25 SPCSV-seropositive wild plants belonging to eight species were grafted onto *I*. *setosa*. The grafted plants developed chlorotic mottling symptoms on leaves and tested SPCSV-positive by TAS-ELISA at 4 wk post-grafting. No plants of *I*. *aquatica* (12 plants tested), *I*. *crepidiformis* (12), *I*. *hilderbrandtii* (6), *I*. *purpurea* (14), *I*. *repens* (3), *A*. *grantii* (17) or *A*. *hyoscyamoides* (14) tested positive for SPCSV (Table 2).

**Table 2:** Incidence of *Sweet potato chlorotic stunt virus* in wild plants and cultivated sweetpotato plants from different agro-ecological zones of Uganda.

			Central zone^d^				Northern zone^d^		Eastern zone^d^						Western zone^d^					
Plant species	Life cycle^a^	Total no. of plants^c^	MKN	MSK	RKI	MPG	GUL	ARU	KTK	SOR	MBL	KAP	TOR	KML	RUK	KNG	BUS	MBR	MAS	HOM
*Ipomoea acuminata*	P	102(7.8)	0(11)	1(9)	-	1(22)	-	-	-	-	4(29)	-	-	-	-	0(17)	1(4)	0(5)	-	1(5)
*I*. *aquatica*	P	12(0)	-	-	-	-	-	-	0(10)	0(2)	-	-	-	-	-	-	-	-	-	-
*I*. *cairica*	P	121(6.6)	0(3)	0(7)	2(20)	1(15)	-	-	-	-	0(3)	-	0(5)	1(14)	0(5)	-	1(19)	1(26)	1(3)	1(1)
*I*. *crepidiformis*	P	12(0)	-	-	-	-	-	-	-	0(12)	-	-	-	-	-	-	-	-	-	-
*I*. *hederifolia*	A	34(8.8)	0(11)	0(5)	-	-	-	-	-	-	-	-	-	2(8)	-	-	-	-	-	1(10)
*I*. *hildebrandtii*	P	6(0)	-	-	0(2)	-	-	-	-	-	0(3)	-	-	-	-	-	-	0(1)	-	-
*I*. *obscura*	P	75(9.3)	1(5)	1(10)	0(8)	0(6)	-	-	-	2(10)	-	1(14)	-	-	-	-	0(6)	1(10)	0(1)	1(5)
*I*. *purpurea*	A	14(0.0)	-	0(6)	0(2)	-	-	-	-	-	-	-	-	-	-	-	-	0(6)	-	-
*I*. *repens*	P	3(0)	-	-	-	-	-	-	-	-	0(3)	-	-	-	-	-	-	-	-	-
*I*. *rubens*	P	10(10.0)	-	0(2)	-	-	-	-	0(6)	1(1)	-	-	0(1)	-	-	-	-	-	-	-
*I*. *sinensis*	A	231(5.6)	-	-	-	-	0(54)	1(36)	1(21)	1(25)	1(7)	-	2(16)	3(23)	-	0(7)	-	-	3(40)	1(2)
*I*. *spathulata*	P	27(3.7)	-	-	-	-	-	-	-	-	-	1(27)	-	-	-	-	-	-	-	-
*I*. *stenobasis*	P	10(10.0)	-	-	-	-	-	1(7)	-	-	0(3)	-	-	-	-	-	-	-	-	-
*I*. *tenuirostris*	P	165(5.5)	1(11)	-	-	1(10)	-	-	-	-	1(17)	2(44)	-	-	1(39)	0(4)	2(32)	1(8)	-	-
*I*. *wightii*	P	51(5.9)	-	0(1)	1(19)	0(1)	-	-	-	-	-	-	-	-	1(5)	0(4)	0(5)	1(14)	-	0(2)
*Astripomoea granti*	P	17(0)	-	0(7)	-	-	-	-	-	-	-	-	-	-	-	0(10)	-	-	-	-
*A*. *hyoscyamoides*	P	14(0)	-	-	-	-	-	-	-	0(5)	-	-	-	0(5)	0(4)	-	-	-	-	-
*Hewittia sublobata*	P	267(2.6)	0(9)	0(28)	0(21)	0(14)	0(2)	-	-	0(11)	0(12)	-	2(34)	1(21)	0(5)	0(18)	0(19)	0(13)	2(19)	2(41)
*Lepistemon owariensis*	P	53(9.4)	0(10)	-	-	0(3)	-	-	-	0(2)	0(3)	-	2(18)	0(12)	-	1(1)	-	-	1(1)	1(3)
Total no. of wild plants^b^		1224(5.4)	2(60)	2(75)	3(72)	3(71)	0(56)	2(43)	1(37)	4(68)	6(80)	4(85)	6(74)	7(83)	2(58)	1(61)	4(85)	4(83)	7(64)	8(69)
SPCSV incidence (%)			3.3	2.7	4.2	4.2	0	4.7	2.7	5.9	7.5	4.7	8.1	8.4	3.4	1.6	4.7	4.8	10.9	11.6
Sweetpotato	P	419(25.1)	4(19)	13(40)	7(28)	5(23)	0(14)	1(21)	2(14)	6(17)	6(33)	2(23)	3(16)	5(32)	10(26)	12(36)	10(21)	7(15)	5(16)	7(25)

^a^Lifecycle of species: A, annual; P, perennial.

^b^Number of SPCFV-positive plants followed (in parentheses) by total number of plants tested per district. Plants were tested for SPCSV only in 2007.

^c^Total number of plants tested per species followed (in parentheses) by percentage of plants of each species testing positive for SPCSV.

^d^Number of SPCSV-positive plants followed (in parentheses) by number of plants tested per district. ‘─’ indicates that the plant species was not observed in that district. Central region districts (Lake Victoria basin): MKN = Mukono, MSK = Masaka, RKI = Rakai, MPG = Mpigi. Northern region districts: GUL = Gulu, ARU = Arua. Eastern region districts: KTK = Katakwi, SOR = Soroti, MBL = Mbale, KAP = Kapchorwa, TOR = Tororo, KML = Kamuli. Western region districts: RUK = Rukungiri, KNG = Kanungu, BUS = Bushenyi, MBR = Mbarara, MAS = Masindi, HOM = Hoima.

Among all 1224 wild plants tested, SPCSV was more frequently detected in plants from the western (1.6–11.6%) and eastern (2.7–8.4%) zones than the central (2.7–4.2%) zone (Table 2). Only two plants from Arua, one of the two northern districts, tested positive for SPCSV (Table 2). Six species (*I*. *acuminata*, *I*. *cairica*, *I*. *obscura*, *I*. *tenuirostis*, *H*. *sublobata* and *L*. *owariensis*) were commonly found in the eastern, central and western zones and were therefore sampled in the largest numbers (64% of all plants tested). Comparison of virus incidence across these three agro-ecological zones confirmed the aforementioned spatial differences in SPCSV incidence (Table 2). SPCSV was also detected in 105 (25%) of the 419 cultivated sweetpotato plants tested.

### Mixed viral infections in wild plants

Mixed infections of SPCFV with any of the three other common sweetpotato viruses, SPCSV, SPFMV and SPMMV, was not found in the wild plants tested. However, co-infections involving the other three viruses were found, including SPCSV + SPFMV and SPCSV + SPMMV in six plants each out of 1224 plants sampled in 2007. Co-infection of SPFMV and SPMMV was detected in 35 of 2864 plants sampled in 2004 and 2007. Triple infection by SPCSV, SPFMV and SPMMV was found in 5 of 1224 plants sampled in 2007 (Table 3). Single infections by SPFMV and SPMMV in wild plants have been reported elsewhere [23, 57].

**Table 3:** Occurrence of mixed infections with SPCFV, SPCSV, SPFMV and/or SPMMV in wild Convolvulacea species and cultivated sweetpotato plants collected in Uganda.

		Virus combinations^d^											
Plant species^a^	Life cycle^c^	CF+FM	CF+CS	CF+MM	FM+MM^e^	FM+MM^f^	FM+MM^g^	CS+FM	CS+MM	CS+FM+MM	CS+FM+CF	CS+MM+CF	CS+FM+MM+CF
*Ipomoea acuminata*	P	-	-	-	3(55)	1(102)	4(157)	1(102)	0(102)	1(102)	-	-	-
*Ipomoea cairica*	P	-	-	-	2(99)	2(121)	4/220	2(121)	0(121)	2(121)	-	-	-
*Ipomoea hederifolia*	P	-	-	-	1(13)	0(34)	1(47)	0(34)	0(34)	0(34)	-	-	-
*Ipomoea obscura*	P	-	-	-	1(79)	0(75)	1(154)	0(75)	2(75)	1(75)	-	-	-
*Ipomoea repens*	P	-	-	-	0(24)	0(3)	0(27)	1(3)	0(3)	0(3)	-	-	-
*Ipomoea sinensis*	A	-	-	-	6(143)	2(231)	8(374)	3(231)	0(231)	1(231)	-	-	-
*Ipomoea spathulata*	P	-	-	-	0(15)	0(27)	0(42)	0(27)	1(27)	0(27)	-	-	-
*Ipomoea stenobasis*	P	-	-	-	0(13)	0(10)	0(23)	1(10)	0(10)	0(10)	-	-	-
*Ipomoea tenuirostris*	P	-	-	-	5(230)	2(165)	7(395)	3(165)	0(165)	0(165)	-	-	-
*Ipomoea wightii*	P	-	-	-	0(62)	0(51)	0(113)	1(51)	0(51)	0(51)	-	-	-
*Hewittia sublobata*	P	-	-	-	6(420)	1(267)	7(687)	2(267)	2(267)	0(267)	-	-	-
*Lepistemon owariensis*	P	-	-	-	2(44)	1(53)	3(97)	2(53)	1(53)	0(53)	-	-	-
Total mixed-infected wild plants^b^		-	-	-	27(1640)	8(1224)	35(2864)	16(1224)	6(1224)	5(1224)	-	-	-
Sweetpotato	P	3(419)	3(419)	2(419)	n/a	9(419)	n/a	60(419)	15(419)	5(419)	2(419)	1(419)	1(419)

^**a**^Lifecycle of species: A, annual; P, perennial.

^**b**^CF = SPCFV, CS = SPCSV, FM = SPFMV, MM = SPMMV.

^**c**^All values represent number of co-infected plants followed (in parentheses) by number of plants tested. ‘─’ indicates that the viral combination was consistently not found in the wild species but was detected in one or more sweet potato plants. n/a = not applicable.

^**d**^The total number of wild plants tested was 1640 in 2004 and 1224 in 2007 (2864 overall). Virus combination FM+MM was detected in 2004 and 2007; all other virus combinations were detected only in 2007. CS was surveyed only in 2007. No co-infections involving CF were detected.

^**e,f,g**^FM + MM data are shown for 2004^**e**^, 2007^**f**^ and 2004+2007^**g**^.

In 419 sweetpotato plants tested, SPCFV was found co-infecting with SPFMV (3 plants), SPCSV (3 plants) or SPMMV (2 plants), and with both SPFMV and SPCSV (2 plants), SPFMV and SPMMV (9 plants) or SPMMV and SPCSV (1 plant). All four viruses were detected in one sweetpotato plant (Table 3).

### Molecular variability of the SPCFV coat protein (CP) and nucleic acid–binding protein (NaBP) regions

The (+)ssRNA genome of SPCFV (NCBI acc. no. AY461421) is 9104 nucleotides (nt) long, excluding the 3′-terminal poly(A) tail, and contains six open reading frames (ORFs) [67]. ORF5 encodes the coat protein (CP). ORF6 partially overlaps the 3′ end of ORF5 by 17 nt and encodes the nucleic acid–binding protein (NaBP) [67] implicated in suppression of antiviral RNA silencing [68]. The length of RT-PCR amplicons covering the 3′-proximal genomic region of SPCFV from five wild plants and four sweetpotato plants was 1578 nt. BLAST searches in the NCBI database showed that the sequences were homologous to the 3′ genomic region in the 29 SPCFV isolates previously characterized from sweetpotatoes in East Africa (Uganda, Kenya, Tanzania), Asia (China, Taiwan, South Korea), Australia, East Timor, Peru or of unknown origin (Table 4). The amplified sequences contained the ORFs for SPCFV CP (nt 242–1138, 299 aa) and NaBP (nt 1125–1523, 133 aa), and also the 3′-UTR; (nt 1527–1578). Sequences determined in this study were submitted to the NCBI database under accession numbers EF155967, EF155968 and KR086396–KR086402 (Table 4).

**Table 4:** Geographical origin of *Sweet potato chlorotic fleck virus* isolates used for comparison of their 3′-terminal genomic regions.

Isolate^a^	Geographical origin	Genebank accession no.	Host	Reference
4MBL	Mbale, Uganda	EF155967	*I*. *acuminata*	This study
BUSH42	Bushenyi, Uganda	KR086399	*I*. *acuminata*	This study
KINT2	Mpigi, Uganda	EF155968	*I*. *cairica*	This study
MAS53	Masindi, Uganda	KR086397	*I*. *tenuirostris*	This study
MBL86	Mbale, Uganda	KR086400	*I*. *acuminata*	This study
ARU91	Arua, Uganda	KR086402	sweetpotato	This study
HOM40	Hoima, Uganda	KR086398	sweetpotato	This study
KNG92	Kanungu, Uganda	KR086401	sweetpotato	This study
RKI15	Rakai, Uganda	KR086396	sweetpotato	This study
007VIIMS	Unknown	EU375897	sweetpotato	[54]
94-1s	Kenya	EU375900	sweetpotato	[54]
AusCan	Australia	EF990647	sweetpotato	[63]
**AusCan**	Australia	KU707475	sweetpotato	[66]
B-Guangdong-11-5	China	KC130184	sweetpotato	Qiao et al., 2012, unpublished
B-Guangxi-11-1	China	KC130186	sweetpotato	Qiao et al., 2012, unpublished
B-Jiangxi-11-4	China	KC130185	sweetpotato	Qiao et al., 2012, unpublished
G-Sichuan-10-60	China	KC130183	sweetpotato	Qiao et al., 2012, unpublished
Gwangzhu1	China	EU375901	sweetpotato	[54]
**HG176**	South Korea	KP715159	sweetpotato	Kwak et al., 2015, unpublished
**HN83**	South Korea	KP115605	sweetpotato	Kwak et al., 2014, unpublished
Hoima3c	Hoima, Uganda	EU375902	sweetpotato	[54]
KBL38	Kabale, Uganda	EU375903	sweetpotato	[54]
Kiboga6b	Kibonga, Uganda	EU375908	sweetpotato	[54]
KY5	Kenya	EU375904	sweetpotato	[54]
Le-97-598	Unknown	EU375905	sweetpotato	[54]
Mas	Masindi, Uganda	AJ781295	sweetpotato	Mukasa et al., 2004, unpublished
Mpigi6b	Mpigi, Uganda	EU375906	sweetpotato	[54]
Njoro5	Kenya	EU375910	sweetpotato	[54]
Rukungiri1b	Rukungiri, Uganda	EU375907	sweetpotato	[54]
**SC20**	South Korea	KP115606	sweetpotato	Kwak et al., 2014, unpublished
SH1	China	KC414676	sweetpotato	[65]
**SPCFV**	Hoima, Uganda	AY461421	sweetpotato	[67]
SPCFV-CIP	Peru	EU375899	sweetpotato	[54]
Tar	Tarime, Tanzania	AJ781296	sweetpotato	Mukasa et al., 2004, unpublished
**Tm37**	East Timor	KU720565	sweetpotato	[66]
TN340	Taiwan	EU375898	sweetpotato	[54]
TN399	Unknown	EU375909	sweetpotato	[54]
**UN210**	South Korea	KP115607	sweetpotato	Kwak et al., 2014, unpublished

^a^ Names of seven SPCFV isolates whose complete genome sequences are currently available are shown in bold.

The nucleotide sequences of the nine SPCFV isolates were 86.1–98.2% (CP; S2 Table), 95.2–99.5% (NaBP; S3 Table) and 96.4–100% (3′-UTR; S4 Table) identical. The five isolates from wild plants were 86.2–98.2% (CP; S2 Table), 95.5–99.2% (NaBP; S3 Table), and 96.4–100% (3′-UTR; S4 Table) identical at the nucleotide level with 15 isolates from cultivated sweetpotato in East Africa. Among all 38 CP- and 32 NaBP-coding sequences of SPCFV, including those determined in this study and those available in the NCBI database, the nucleotide sequence identities were 75.0–100% for the CP (S2 Table) and 77.4–100% for the NaBP (S3 Table), and the deduced amino acid sequence identities were 88.3–100% for the CP (S2 Table) and 75.9–100% for the NaBP (S3 Table). However, identities between SPCFV isolates from East Africa and elsewhere were relatively low: 75.0–89.3% and 88.3–95.7% at the nucleotide and amino acid level, respectively, for CP (S2 Table), and 77.4–93.7% and 75.9–96.2% at the nucleotide and amino acid level for NaBP (S3 Table). NaBP of SPCFV is a cysteine-rich protein (CRP) [68] and has a zinc finger–like motif (CX_2_CX_4_CX_3_C) that was observed within the same protein region (aa 64–98) in all nine SPCFV isolates. The arginine-rich basic motif, RRARR, which is involved in the RNA silencing suppression activity of NaBP [68] was also observed in the same position (aa 59–63) in all nine SPCFV isolates. The CP of isolates BUSH42 and KINT2 from wild plants had unique amino acid substitutions (V/G/E/S12A and Q119H, respectively), and the NaBP of KINT2 had a unique amino acid substitution (I34V). Some amino acid sites in the CP (13E, 29E, 41I and 118A) and NaBP (3S, 7R, 23C, 74E and 95V) were conserved in isolates from East Africa but were highly variable in isolates from Asia. Overall, no consistent amino acid sequence differences were associated with geographic origin or host species.

### Recombination and phylogenetic relationships in SPCFV isolates

No evidence for recombination was detected in the 1109–1761 nt-long NaBP-CP-3′-UTR region available from 35 SPCFV isolates (*P* = 0.999) or in the complete genomic sequences of the seven SPCFV isolates indicated in Table 4 (*P* = 0.071) using the six programs included in the RDP4 package and the PHI test.

**Fig 3:**
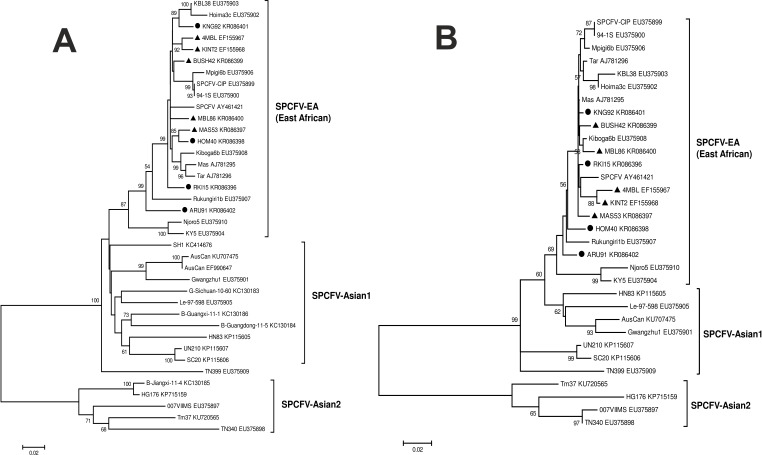
Phylogenetic analysis of SPCFV based on the CP and NaBP nucleotide sequences. Nine SPCFV isolates from wild plant species (▲) or sweetpotatoes (●) in this study are compared with 29 and 23 isolates, respectively, from previous studies. (**A, B**) Sequences for CP (**A**) and NaBP (**B**) were analyzed. Sequences cluster according to the geographical origin of the virus isolates, i.e., East Africa (SPCFV-EA) or Asia (SPCFV-Asian1 and SPCFV-Asian2). The geographical origins are unknown for isolates Le-97-598_EU375905, TN399_EU375909 and 007VIIMS_EU375897. Numbers at branches represent bootstrap values of 1000 replicates. Only bootstrap values of ≥50% are shown. Scale indicates nucleotide substitutions per site according to Tamura [69].

Using the T92+G+I nucleotide substitution model, phylogenetic clustering of the 38 CP sequences showed no congruence with the host species (Fig 3A). However, there was phylogenetic congruence of isolates according to their geographic origin in East Africa and Asia. All isolates from East Africa (including Uganda, Kenya and Tanzania) were designated as SPCFV-EA (Fig 3A, Table 4). Isolates from Asia were clustered into two groups, designated as SPCFV-Asian1 (comprising isolates from Australia, China, South Korea and Taiwan or of unknown origin) and SPCFV-Asian2 (comprising a few isolates from China, Taiwan and East Timor or of unknown origin) (Fig 3A, Table 4). An exception was isolate SPCFV-CIP (accession no. EU375899) from Peru, which clustered with isolates from East Africa (Fig 3A). Phylogenetic clustering of isolates based on 32 NaBP nucleotide sequences (using the substitution model T92+G) was similar to that of CP (Fig 3B). The 3′-UTR sequences were too short (52 nt) for meaningful analyses and were not included in phylogenetic analyses.

### Nucleotide diversity and selection pressure on the SPCFV CP and NaBP

Analysis of genetic differentiation between SPCFV populations from East Africa and Asia was carried out for both the CP- and NaBP-coding sequences. *F*_ST_ values for CP (0.30011) and NaBP (0.30064) showed evidence of genetic differentiation, implying that for each of the CP and NaBP, 30.0% of total variance of the SPCFV population is explained by the origin of isolates in East Africa or Asia. Between-population diversity was greater than within-population diversity for CP and NaBP, further suggesting a differentiated population. For example, the SPCFV subpopulation from East Africa has a within-population diversity of 0.05007 ± 0.00590 (CP) and 0.02934 ± 0.00482 (NaBP). However, between-population diversities with the Asian subpopulation was more than two times higher, both separately (0.11003 ± 0.00751, Asian1 CP; 0.11405 ± 0.01952, Asian2 CP; 0.08593 ± 0.00886, Asian1 NaBP; and 0.07018 ± 0.001704, Asian2 NaBP) or in combination (0.15861 ± 0.01220 for the CP and 0.14723 ± 0.01336 for the NaBP). In contrast, the SPCFV subpopulation from outside East Africa had within-population diversities of 0.15861 ± 0.01220 (CP) and 0.14723 ± 0.01336 (NaBP), which are only slightly higher than the between-population diversities, indicating a subpopulation structuration in the Asian isolates. Taken together, the phylogenetic clustering of isolates (Fig 3A and 3B), gene flow estimates of *F*_ST_ and within- and between-population diversity indices demonstrate genetic differentiation of SPCFV according to geographical origin.

Synonymous codon usage bias was evaluated based on the effective number of codons (ENC). For the nuclear universal genetic code, the value for ENC ranges from 20 (if only one codon is used for each amino acid, i.e., codon bias is maximal) to 61 (if all synonymous codons for each amino acid are equally used, i.e., there is no codon bias). Our results showed that CP had a higher ENC value (53.9) than NaBP (50.1), suggesting that, although both coding regions had moderate bias in codon usage, NaBP had more codon bias than CP. This is consistent with the larger codon bias index (CBI) value found for NaBP (0.432) as compared with CP (0.283). CBI values range from 0 (in a gene with random codon usage) to 1 (in a gene with extreme codon bias). Thus, our CBI results suggest that codon usage is more random in CP than in NaBP.

Irrespective of the host species from which the SPCFV isolates were characterized, nucleotide diversity (π) values for each of the two protein-coding regions were relatively low (12.1% and 8.7% for *CP* and *NaBP*, respectively). The non-synonymous nucleotide diversity (π_a_) was 2.6% and 4.1% for *CP* and *NaBP*, respectively, whereas the synonymous nt diversity (π_s_) was 45.7%, and 25.2%, respectively. The ratio of π_a_ to π_s_ (ω = π_a_/π_s_) gives a generalized estimation of ω, which is the measure of selection pressure imposed on a given entire protein. The value of π_a_ was 17.5- and 6.1-fold lower than the value of π_s_ for *CP* and *NaBP*, respectively, suggesting the influence of purifying selection. Under the basic assumption that a codon is a unit of evolutionary change [70], maximum likelihood (ML) site models treat ω for any codon in a protein-coding nucleotide sequence as a random variable from a statistical distribution. Thus, selection pressures suggested by the aforementioned results and assessed by a ML framework of codon substitution under model M0, which yielded ω values of 0.044 and 0.127 for *CP* and *NaBP*, respectively, indicate purifying selection (Table 5). The heterogeneity of selective pressure was revealed by a likelihood ratio test (LRT) of M3 vs. M0, which showed that the M3 model fit the data significantly better than M0 for both CP and NaBP proteins (Table 5). M3 for NaBP suggested that 58.0% of sites were subject to strong purifying selection (ω = 0.011), 40.5% of sites were under weak purifying selection (ω = 0.278) and only 1.4% of sites were under positive selection (ω = 1.598) (Table 5). Näive empirical Bayes inference under M3 identified one amino acid (8P) as undergoing positive selection (Table 5). M3 for CP showed that all sites were under varying degrees of purifying selection as follows: 82.7% of sites were subjected to nearly lethal mutations (ω = 0.008), 14.5% were under weak purifying selection (ω = 0.177) and 2.7% of sites were under nearly neutral evolution (ω = 0.615) (Table 5). In both CP and NaBP, likelihood ratio tests (LRTs) of nested models M2a vs. M1a, M8 vs. M7 and M8a vs. M8 showed that the positive selection models (M2a, M8 and M8a) did not fit the data significantly better than the respective null models (M1a, M7 and M8; Table 5), which is consistent with purifying selection on many of the amino acid sites. Parameter estimates under each of the models are shown in Table 5.

**Table 5:** Parameter estimates, log-likelihood (*ln*L), *ω*-ratio (*d*_N_/*d*_S_), and likelihood ratio test (LRT) statistics under seven different maximum likelihood models of codon substitution used to investigate selection pressures exerted on NaBP and CP of SPCFV.

Protein	Model^a^	Parameter estimates^b^	ω-ratio (d_N_/d_S_)	Log likelihood (*ln*L)	LRT statistic^c^ (2×δ*ln*L)	Positively selected (amino acids) sites^d^
CP	M0	ω = 0.044	0.044	−6692.464		none
	M3	*p*_0_ = 0.827, *p*_1_ = 0.145 (*p*_2_ = 0.027); ω_0_ = 0.008, ω_1_ = 0.177, ω_2_ = 0.615	0.049	−6562.318	*p* < 0.0001 (M0 vs. M3)	none
	M1a	*p*_0_ = 0.943 (*p*_1_ = 0.056), ω_0_ = 0.024 (ω_1_ = 1.000)	0.079	−6615.790		not allowed
	M2a	*p*_0_ = 0.943, *p*_1_ = 0.056 (*p*_2_ = 0.000); ω_0_ = 0.024, ω_1_ = 1.000, ω_2_ = 35.321	0.079	−6615.790	*p* > 0.05 (M1a vs. M2a)	none
	M7	*p* = 0.136, *q* = 2.240	0.052	−6564.566		not allowed
	M8	*p*_0_ = 0.986 (*p*_1_ = 0.014); *p* = 0.155, *q* = 3.305, ω_s_ = 1.000	0.053	−6563.764	*p* > 0.05 (M7 vs. M8)	none
	M8a	*p*_0_ = 0.981 (*p*_1_ = 0.018); *p* = 0.160, *q* = 3.708, ω_s_ = 0.800	0.052	−6562.619	*p* > 0.05 (M8 vs. M8a)	none
NaBP	M0	ω = 0.127	0.127	−2060.857		none
	M3	*p*_0_ = 0.580, *p*_1_ = 0.405 (*p*_2_ = 0.014); ω_0_ = 0.011, ω_1_ = 0.278, ω_2_ = 1.598	0.143	−2031.819	*p* < 0.001 (M0 vs. M3)	8P**
	M1a	*p*_0_ = 0.893 (*p*_1_ = 0.107), ω_0_ = 0.077 (ω_1_ = 1.000)	0.176	−2039.934		not allowed
	M2a	*p*_0_ = 0.893, *p*_1_ = 0.074 (*p*_2_ = 0.033); ω_0_ = 0.078, ω_1_ = 1.000, ω_2_ = 1.000	0.176	−2039.934	*p* > 0.05 (M1a vs. M2a)	none
	M7	*p* = 0.312, *q* = 1.818	0.143	−2033.003		not allowed
	M8	*p*_0_ = 0.991 (*p*_1_ = 0.009); *p* = 0.368, *q* = 2.431, ω_s_ = 1.915	0.144	−2031.822	*p* > 0.05 (M7 vs. M8)	8P
	M8a	*p*_0_ = 0.958 (*p*_1_ = 0.042),; *p* = 0.401, *q* = 3.130, ω_s_ = 0.800	0.139	−2032.608	*p* > 0.05 (M8 vs. M8a)	8P

^a^Models are according to the descriptions given in the methods.

^b^Numbers of parameters for different models were 1 (M0), 2 (M1a), 4 (M2a), 5 (M3), 2 (M7), 3 (M8a) and 4 (M8).

^c^LRT statistics of M3 *vs*. M0 are tests of heterogeneity of selection pressures among codon sites, while those of M2a *vs*. M1a, M8 *vs*. M7 and M8 *vs*. M8a are tests of positive selection, all of which assess the LRT statistic (2δ*ln*L) against a chi-square distribution with degrees of freedom (d.f.) equal to the difference in the number of parameters between the nested models under comparison.

^d^A positively selected amino acid site with posterior probability *P* > 99 (**) is shown. Identification of amino acid under positive selection is based on näive empirical Bayes (NEB) (under M3) or Bayes empirical Bayes (BEB) inference (under M2a, M8 or M8a).

## Discussion

Most of the 26 tested wild species of Convolvulaceae were found to be natural hosts for SPCFV, including *H*. *sublobata*, *L*. *owariensis* and 15 *Ipomoea* species. Previously, *I*. *aquatica*, *I*. *purpurea* and *I*. *wightii* were shown to be infectible with SPCFV following experimental inoculation [54], but this study showed that these species can be naturally infected with SPCFV. Furthermore, SPCSV was found to infect 12 species in the field, including *H*. *sublobata*, *L*. *owariensis* and 10 wild *Ipomoea* species. These results significantly extend our knowledge of the natural host ranges of SPCSV and SPCFV.

Many of the wild plants tested contained double or triple infections of SPFMV, SPMMV and/or SPCSV. These mixed infections have not been reported previously. However, no wild species were co-infected with SPCFV and any of the other three viruses. This was in striking contrast to cultivated sweetpotatoes, which are frequently co-infected with SPCFV and one or more of the other viruses both in our analysis and in previous studies in East Africa [53, 58, 61]. Furthermore, our previous studies have shown that several wild Convolvulaceae species are co-infected with the SPFMV strains EA and C in the field in Uganda [22]. The C strain was proposed to be a new species [71] and was recently designated as *Sweet potato virus C* [72]. In sweetpotatoes, the incidence of SPFMV and SPCSV infections can be as high as 70% in Uganda [58], which in turn increases the incidence of co-infection, development of SPVD and significant yield losses.

Perennial host plants and generalist vectors of viruses could be expected to enhance mixed infection. In East Africa including Uganda, the perenniality of sweetpotato in the local cropping system favours accumulation of viruses and mixed infections are common [42, 46]. Also, mixed virus infections are known in perennial wild plants, e.g., [13, 73–75]. However, whether high incidence of mixed virus infections could be linked to the plants’ perennial or annual lifecycle requires further study. For example, an annual grass species with less resistance to virus infection showed a high potential of acting as a reservoir of a generalist plant virus that also infects perennial grass hosts growing in the same habitat [2, 76–79]. Furthermore, co-infection by a group of vectored viral pathogens is highest with abundant generalist vectors (which are able to transmit multiple virus species/strains), weak cross‐protection and co-infection–induced mortality [75, 80]. Although it is known that aphids transmit SPFMV, and whiteflies (*Bemisia tabaci* and *Trialeurodes abutilonea*) transmit SPCSV, the vectors for SPCFV and SPMMV remain to be confirmed [55]. This currently limits our ability to elucidate the impact of vectors on the contrasting incidences of mixed viral infections in wild species and sweetpotatoes. However, cross-protection between any of the virus species in our study is unlikely, because it requires high sequence homology [81]. Therefore, the most probable explanation of our observed low incidences of mixed infections may be inefficient vector transmission of viruses between the wild plants or between cultivated and wild plants [31] and/or high levels of virus resistance in wild species preventing infection or keeping virus titers at undetectable levels [12, 82]. Furthermore, synergistic or additive effects of multiple virus infections causing severe disease could have eliminated co-infected plants [1–2, 5, 83–86]. These effects can vary among populations [12, 87, 88], species [89] and environments [75, 90, 91].

Contrasting virus incidences in wild plants may be explained by community contexts and processes [92, 93]. For example, in the luteovirus complex (barley and cereal yellow dwarf viruses) in California grasslands, virus prevalence is shaped by interactions within the plant community and among host plants, insect vectors, herbivores and abiotic factors [92, 94–96]. Although general differences in natural vegetation types have been previously noted in Uganda [57, 58], empirical data on host plant community composition needs to be strengthened to warrant testable hypotheses on contrasting regional virus incidences in wild plants.

Observation of disease symptoms is the initial step in viral disease diagnosis. Although virus-like symptoms were observed, no characteristic symptoms could be associated with a particular virus for several reasons, including mixed infections and condition of the host. Furthermore, many SPCFV- and SPCSV-infected wild plants remained symptomless, which seems common among wild plants [5, 8, 13, 18, 89, 97]. In addition, some symptom-expressing plants tested negative for SPCFV, SPCSV, SPMMV and SPFMV, indicating possible infection with other viruses that could not be detected with the antibodies and PCR primers used due to assay specificities. It seems worthwhile to continue these studies using generic methods, such as small-RNA deep sequencing, that require no presumptions about the viruses present and can detect all types of viruses simultaneously [98–102].

The *CP* and *NaBP* sequences of five SPCFV isolates from three wild host species and their comparison with 11 SPCFV isolates from cultivated sweetpotato in Uganda revealed nearly identical nucleotide diversity indices and no phylogenetic evidence of diversification because of the host species. Negative selection was implicated in the evolution of CP and NaBP. Negative constraints imposed by mutations on viral CPs may be associated with multiple functions such as genome encapsidation and protection, cell-to-cell movement, transmission between plants and host and/or vector interactions. Chare and Holmes [103] analyzed selection pressures in CP-coding sequences of plant RNA viruses and found that vector-borne viruses are subjected to greater negative selection than non-vectored viruses. Negative selective pressure is usually interpreted as a mechanism of preserving the structure and function of proteins [70, 104]. The CP of SPCFV and other carlaviruses is multifunctional [104–106], whereas NaBP is a cysteine-rich protein (CRP) implicated in RNA silencing suppression, nuclear localization and viral pathogenesis [68, 107–109]. In NaBP and CP, different codon positions were subjected to varying levels of purifying selection, possibly to provide a balance between the need to maintain protein structure and function and the effectiveness of these functions. The lack of a CRP in the sweet potato C-6 carlavirus (SPC6V) [110] may also indicate that CRPs are to some extent redundant in carlaviruses.

Most of the wild plants in this study were collected from the vicinity of sweetpotato fields or grew as weeds in sweetpotato fields. This makes it easier for putative vectors to transmit viruses between wild and cultivated hosts. Indeed, the observed similarities and lack of phylogenetic congruence with wild and cultivated hosts suggests frequent exchange of SPCFV isolates between the wild plants and sweetpotatoes. Similarly, no phylogenetic association with any hosts has been found in three other carlavirus species (*Shallot latent virus*, *Garlic latent virus* and *Common garlic latent virus*) infecting six different *Allium* spp. [111]; isolates of SPMMV, SPFMV and SPCSV in Uganda [22–24]; *Rice yellow mottle virus* (genus *Sobemovirus*) in cultivated rice and wild graminaceous species in East, Central and West Africa [28, 112] and *African cassava mosaic virus* and *East African cassava mosaic Cameroon virus* (genus *Begomovirus*, family Geminiviridae) in cassava and various wild hosts in West Africa [113].

Phylogenetic clustering of SPCFV isolates was congruent with their geographic origin in East Africa or Asia, demonstrating diversification. This has also been shown for several other economically harmful viruses infecting sweetpotato, cassava or rice, suggesting that East Africa is a center of evolutionary diversification and emergence of many new plant viruses and virus strains. For example, the East African (EA) strain of SPFMV (SPFMV-EA) is mainly found in East Africa [22, 71, 114–117], where it is undergoing rapid molecular adaptation compared with other strains of SPFMV and *Sweet potato virus* C (SPVC) [22]. Until recently, an EA strain of SPCSV (SPCSV-EA) was restricted to East Africa. The SPCSV-EA isolates vary in the presence or absence of a coding region for a p22 RNA silencing suppressor, whereas SPCSV isolates from outside East Africa typically lack the p22 [24, 42, 118–120]. SPMMV is geographically restricted to East Africa [23, 71, 121], in contrast to SPCFV, which is found on many continents. Preliminary evidence suggests that SPCFV isolates from East Africa may be distinguished from those occurring elsewhere by phylogenetic analysis of *CP* sequences [54]. However, the inclusion of additional SPCFV isolates from East Africa and analysis of *CP* and *NaBP* sequences in this study clearly showed that SPCFV isolates from East Africa form a unique phylogenetic group. Hence, we propose the name SPCFV-EA for the strains typical of East Africa.

Other plant viruses also seem to have a center of diversification in East Africa. *Cassava brown streak virus* and *Ugandan cassava brown streak virus* occur in East Africa, where they have a modular distribution in Indian Ocean coastal areas and the mainland Lake Victoria basin [122–126]. However, they are now spreading to other areas [127, 128]. Cassava mosaic geminiviruses, including a highly virulent recombinant strain, exhibit a gradient of decreasing prevalence (100% to 38%) from eastern to southern Africa [129, 130]. *Rice yellow mottle virus* exhibits phylogenetic congruence with the geographical origin of isolates on an east-to-west transect across Africa and showed decreased nucleotide diversity westward across Africa [28, 112, 131, 132]. The recently emerged strain S4ug of the virus in Eastern Uganda is thought to be the outcome of singular interplay between strains in East Africa and Madagascar [133]. Although there are relatively few characterized isolates of SPCFV (*n* = 38), the strong phylogenetic affinity to their origin in East Africa is another piece of evidence implicating East Africa as a hot spot for diversification of important plant viruses.

Taken together, the current study further highlights wild plants as reservoirs of viruses in agro-ecosystems. The four viruses detected in wild Convolvulaceae plants in Uganda cause major constraints in sweetpotato production in East Africa. Symptomless viral infections in wild plant species were common, which is typical of viruses in wild plants and reflects adaptation [8–10, 97]. Plant viruses and their principal hosts often have common centers of origin [134–136]. The sweetpotato originated in Central and/or South America and was dispersed to Africa and other continents only during the last 300 years, although there is evidence of prehistoric cultivation in Australasia and the South Pacific [137–142]. If viruses had been dispersed along with the sweetpotato, it would be expected that identical isolates of SPFMV, SPCSV, SPMMV and SPCFV would occur worldwide. This seems to be the case for SPFMV strains RC, O and C (SPVC), but apparently not for SPFMV-EA, SPCSV-EA or SPCFV-EA, which are largely geographically confined to East Africa [22, 24, 57, 58, 71, 114, 143, 144], this study. The origin of SPMMV is likely to be East Africa, and the sweetpotato is probably not its primary host [23]. Hence, it seems that these sweetpotato viruses are undergoing unique processes of evolution and adaptation in sweetpotato landraces and wild Convolvulaceae species in East Africa.

## Materials and Materials

### Field surveys and sampling

Wild plants (family Convolvulaceae; genera *Astripomoea*, *Ipomoea*, *Hewittia* and *Lepistemon*) including annual, biannual and perennial species were observed for virus symptoms, and a total of 1640 and 1224 plants were collected in the four agro-ecological zones of Uganda (Fig 1) in 2004 and 2007, respectively, as described [57]. All the sampling sites in all zones were on privately owned land and the owners gave gave permission to conduct the study on these sites. The field studies did not involve endangered or protected species. Five to ten leaves (preferably with virus-like symptoms) and two to five cuttings (length, 10–25 cm) were sampled from each plant. Cuttings were planted in an insect-proof screenhouse at the Makerere University Agricultural Research Institute, Kabanyolo (MUARIK), Uganda. The plants studied were mainly in close proximity to sweetpotato cultivation or grew as weeds in sweetpotato fields. Wild plants were identified taxonomically using keys from Verdcourt [145] and by DNA barcoding (accession no. FJ795781-FJ795796) as described [22, 57]. In addition, a total of 419 cultivated sweetpotato plants were sampled from fields in whose vicinity wild plants were collected.

### Serological detection of SPCFV and SPCSV in wild plants

To detect viruses, leaf discs (2 cm in diameter) were excised from 5–10 leaves of a plant, combined and tested by nitrocellulose membrane enzyme-linked immunosorbent assay (NCM-ELISA) using polyclonal antibodies as described [57, 146]. The antibodies were provided by the International Potato Center (CIP), Lima, Peru. All wild plants and sweetpotatoes were tested for SPCFV, but only wild plants and sweetpotatoes sampled in 2007 were tested for SPCSV. Leaf discs were also excised as above for triple antibody sandwich ELISA (TAS-ELISA) for serological testing [147] using polyclonal antibodies specific to the EA strain of SPCSV (antibodies provided by CIP). Testing was repeated on plants established in the screenhouse.

Scions of 25 wild plants seronegative for SPCFV, 40 plants seronegative for SPCSV (but displaying virus-like symptoms) and 30 symptomless plants seronegative for SPCFV and SPCSV were grafted onto 2-wk-old plants of *I*. *setosa* Kerr., a sensitive indicator and nearly universal host of sweetpotato–infecting viruses [148, 149]. The grafted *I*. *setosa* plants were observed for virus symptoms and tested serologically for SPCFV and SPCSV 3 and 4 wk after grafting, respectively, as described above.

The SPCSV isolates detected in wild plants were graft-transmitted to sweetpotato plants of cultivar ‘Tanzania’ for ease of maintenance and further analysis.

### Molecular detection of SPCFV and SPCSV

The presence of SPCFV and SPCSV was verified in 5 and 30 seropositive samples, respectively, by RT-PCR. Total RNA was extracted from 200 mg leaf tissue using TRIzol Reagent (Invitrogen) according to the manufacturer’s instructions. First-strand cDNA was synthesized from 3 μg total RNA using an oligo-dT25 primer (for SPCFV) or random hexamers (for SPCSV) and *Moloney murine leukemia virus* reverse transcriptase RNase H^−^(Finnzymes) according to the manufacturer’s instructions. The cDNA was diluted 10-fold for use in PCR.

The 3′-proximal part of the SPCFV genome (1578 nt according to AY461421), including the CP- and NaBP-coding regions and the 3′-UTR [67], was PCR-amplified using primers designed in this study (forward primer CFVF: 5′-GTCTTTAGR(A/G)TTK(G/T)TR(A/G)AGAY(T/C)TTA-3′; reverse primer CFVR: 5′-GCTCAAAAGTACTTTAAAAC-3′). These primers were complementary to nt 7527–7547 and 9085–9104 in the triple gene block 3 (TGB3) protein-coding sequence and 3′-UTR genomic region of SPCFV (AY461421), respectively. For SPCSV, the 3′ genomic region of RNA1 was amplified using forward primer CSVR3-F2 (5′-GTGTTTCATACATTGTTTGTGTGCT-3′) and reverse primer CSVp22-R2 (5′-AGGTGTATGACTCTAGGGTATAAAC-3′) [24]. The PCR mixture and cycling parameters were those recommended for Phusion High-Fidelity DNA Polymerase (Finnzymes).

PCR products were purified using a combination of exonuclease I (*Exo*I) and calf intestinal alkaline phosphatase (CIAP) (Fermentas) as recommended by the manufacturer. *Exo*I degrades excess primers (ssDNA) and CIAP degrades unincorporated dNTPs, both of which may inhibit the dideoxy PCR sequencing reaction [150]. Purified products from two independent PCRs were sequenced directly in both directions using the Big Dye Terminator kit version 3.1 (Applied Biosystems) on an ABI automatic 3130 XL Genetic Analyzer. The sequences obtained were compared by BLAST search with existing sequences available in the National Center for Biotechnology Information (NCBI) database.

### Multiple sequence alignments and fitting of nucleotide substitution models

Nucleotide sequences were aligned using CLUSTALX version 1.83 [151], examined visually and translated into amino acid sequences using the EMBOSS web translation tool (http://www.ebi.ac.uk/emboss/transeq/index.html). Percent nucleotide and amino acid identities between sequences were computed using the CLUSTALW procedure [152] as implemented in the MEGALIGN program of the DNASTAR software package.

A ML method implemented in MEGA6 [153] was used to find the best nucleotide substitution model explaining the mode of evolution. Models with the lowest Bayesian information criterion (BIC) scores were considered to best describe the substitution pattern.

### Tests for recombination and phylogenetic relationships between SPCFV isolates

The presence of recombination in the sequence data was tested using the pairwise homoplasy index test [154] as implemented in SplitsTree4 version V4.14.2 [155]. Parent-like sequences and approximation of recombination breakpoints were assessed using the RDP, GENECONV, BOOTSCAN, MAXIMUM CHI SQUARE, CHIMAERA and SISTER SCAN methods as implemented in the Recombination Detection Program (RDP4) package [156].

A phylogenetic tree based on CP sequences was constructed using the neighbor joining method [157] and the Tamura three-parameter nucleotide substitution model (T92) [69] with invariant sites and gamma distribution of rates across sites (T92+G+I). Initially, the general time-reversible (GTR) models [158] with invariant sites and gamma distribution of rates across sites (GTR+G+I) or with variable sites (GTR+G) were the most appropriate models for nucleotide substitution for the CP data. However, because of problems associated with implementing the GTR model [159, 160], the T92 model with invariant sites and gamma distribution of rates across sites (T92+G+I) was thus used for the CP, because it provided the next lowest BIC score. For construction of phylogenetic tree based on NaBP sequences, T92 with gamma distribution across sites (T92+G) was used. Both substitution models were deduced by model fitting (above), which allowed modeling of evolutionary rate differences among sites. A bootstrapped consensus tree was inferred from 1000 replicates for each of the above data sets for CP and NaBP. All phylogenetic analyses were implemented using MEGA6 [153].

### Nucleotide diversities and population differentiation in SPCFV

Population genetics parameters with respect to the average number of nucleotide differences between two random sequences in a population (or nucleotide diversity index, π) and the average number of nucleotide substitutions per non-synonymous (π_a_) and synonymous (π_s_) sites were computed. Synonymous codon usage bias was measured by quantifying the codon bias index (CBI) [161] and the effective number of codons (ENC) [162] used in a gene.

The extent of genetic differentiation or level of gene flow between subpopulations was evaluated by estimating *F*_ST_. *F*_ST_ measures the degree of genetic differentiation between two putative subpopulations by comparing the agreement between two haplotypes drawn at random from each subpopulation with the agreement obtained when the haplotypes are taken from the same subpopulation. *F*_ST_ ranges from 0 to 1 for undifferentiated to fully differentiated populations, respectively. Population genetics parameters and gene flow estimates were calculated using DnaSP version 5 [163].

### Analysis of selection pressure on CP and NaBP

The ratio of non-synonymous (*d*_N_) to synonymous (*d*_S_) nucleotide substitution rates (ω = *d*_N_/*d*_S_) provides a sensitive measure of selective constraints at the protein level. Values of ω < 1, ω = 1 and ω > 1 indicate purifying (or negative) selection, neutral evolution and diversifying (or positive) selection, respectively. Based on this, the direction and intensity of selection pressure on a functional protein can be predicted [70, 164]. The maximum likelihood (ML) approach was applied to the CP (38 sequences) and NaBP (32 sequences) used in phylogenetic analysis of SPCFV using seven site models of codon evolution implemented in the CODEML program of the PAML package (version 4.7) [165]. The models used include M0 (one-ratio), M1a (nearly neutral), M2a (positive selection), M3 (discrete), M7 (beta), M8 (beta & ω) and M8a (beta & ω = 1) as described [104, 166, 167]. The probability of observing data was computed as the log likelihood, which is the sum of probabilities over all codons in the sequence. Selection pressure was examined by assessing the value ω and comparing the log likelihoods of nested models (M0 versus M3, M1a versus M2a, M7 versus M8 and M8 vs. M8a) in likelihood ratio tests (LRTs) as described [166, 168]. Where LRTs were significant, a Bayes empirical Bayes inference [167] was used to identify the amino acid(s) under positive selection.

## Supporting Information

S1 Table Number of plants sampled from wild species and cultivated sweetpotato for detection of SPCFV in different agro-ecological zones of Uganda in 2004 and 2007. (DOC)

S2 Table Coat protein (CP) nucleotide sequence (897 nt, upper diagonal) and amino acid sequence (299 aa, lower diagonal) identities (%) of 38 SPCFV isolates. (DOC)

S3 Table Nucleic acid–binding protein (NaBP) nucleotide sequence (399 nt, upper diagonal) and amino acid sequence (133 aa, lower diagonal) identities (%) of 32 SPCFV isolates. (DOC)

S4 Table 3′-untranslated region (3′-UTR) nucleotide sequence identities (%) of 32 SPCFV isolates. (DOC)
