# Supplementary material for: Mixed Infections of Four Viruses, the Incidence and Phylogenetic Relationships of Sweet Potato Chlorotic Fleck Virus (Betaflexiviridae) Isolates in Wild Species and Sweetpotatoes in Uganda and Evidence of Distinct Isolates in East Africa
Source: PLoS One. 2016 Dec 22;11(12):e0167769. doi: 10.1371/journal.pone.0167769 (PMC5179071; doi:10.1371/journal.pone.0167769)
Supplement: S1 Table — (DOC) [file pone.0167769.s001.doc]

**S1 Table.** Number of plants sampled from wild species and cultivated sweetpotato for detection of SPCFV in different agro-ecological zones of Uganda in 2004 and 2007

^a^Lifecycle of species: A, annual; BA, biannual; P, perennial [57, 67].

^b,c^Total number of wild plants^b^; numbers in parentheses represent percentage of plants from a given district^c^ that tested positive for SPCFV in a given year.

^d^Number of SPCFV-positive plants followed (in parentheses) by number of plants of a given species sampled from a given district. ‘─’ indicates that the plant species was not observed in that district or that a district was not surveyed. Central region districts (Lake Victoria basin): LUW = Luwero, MKN = Mukono, MSK = Masaka, RKI = Rakai, MPG = Mpigi. Northern region districts: LIR = Lira, APC = Apac, GUL = Gulu, ARU= Arua. Eastern region districts: KTK = Katakwi, SOR = Soroti, KUM = Kumi, MBL = Mbale, KAP = Kapchorwa, TOR = Tororo, KML = Kamuli, IGG = Iganga. Western region districts: RUK = Rukungiri, KNG = Kanungu, KBL = Kabale, BUS = Bushenyi, MBR = Mbarara, KAS = Kasese, MAS = Masindi, HOM = Hoima.
