# Supplementary material for: Mixed Infections of Four Viruses, the Incidence and Phylogenetic Relationships of Sweet Potato Chlorotic Fleck Virus (Betaflexiviridae) Isolates in Wild Species and Sweetpotatoes in Uganda and Evidence of Distinct Isolates in East Africa
Source: PLoS One. 2016 Dec 22;11(12):e0167769. doi: 10.1371/journal.pone.0167769 (PMC5179071; doi:10.1371/journal.pone.0167769)
Supplement: S3 Table — (DOC) [file pone.0167769.s003.doc]

**S3 Table.** Nucleic acid–binding protein (NaBP) nucleotide sequence (399 nt, upper diagonal) and amino acid sequence (133 aa, lower diagonal) identities (%) of 32 SPCFV isolates

^a^Isolates characterized in this study: (▲), wild plants; (●), sweetpota
